# Exercise on Striatal Dopamine Level and Anxiety-Like Behavior in Male Rats after 2-VO Cerebral Ischemia

**DOI:** 10.1155/2022/2243717

**Published:** 2022-09-13

**Authors:** Yongzhao Fan, Xiaoyang Kong, Kun Liu, Hao Wu

**Affiliations:** ^1^Capital University of Physical Education and Sports, Comprehensive Key Laboratory of Sports Ability Evaluation and Research of the General Administration of Sport of China, Beijing Key Laboratory of Sports Function Assessment and Technical Analysis, Beijing 100191, China; ^2^Brain Peace Science Foundation, CT 06511, USA

## Abstract

The purpose of this study was to discuss the effect of voluntary wheel running on striatal dopamine levels and anxiety-like behavior in rats with global cerebral ischemia. The male Sprague-Dawley rats were signed on in this study and randomly divided into following 4 groups: Control group (C group), Sham group (S group), ischemia group (I group), and 3 weeks physical exercise before ischemia group (3RI group). The rats in the 3RI group were placed in a voluntary running wheel for three weeks to exercise. Then, the rats in I and 3RI groups received bilateral carotid artery ligation (2-VO) operation. The C and S group did not perform voluntary running exercise and the bilateral common carotid arteries of S group were exposed without ligation. In vivo microdialysis was used in conjunction with high performance liquid chromatography (HPLC) and electrochemical detection to ascertain the level of dopamine in the striatum. Elevated plus maze (EPM) and open field (OF) were used to test anxiety status at 24 hours and 7days after 2-VO cerebral ischemia. Meanwhile, gait and motor coordination evaluations were carried out to eliminate the influence of non-specific motor problems. The results indicated that cerebral ischemia instigate the increase of striatal dopamine in I group rats during acute cerebral ischemia. A 3-week voluntary wheel running significantly enhances the striatal dopamine before ischemia and obstructs a further increase of dopamine during acute cerebral ischemia in 3RI group rats. At 24 hours after ischemia, striatal dopamine returned to pre-ischemic levels in 3RI group. Striatal dopamine in I group were less than pre-ischemic levels at 7 days. Behavioral data indicated that 3-week voluntary wheel running promoted recovery of anxiety-like behavior and gait were not affected by 2-VO cerebral ischemia at 24 hours post-ischemia rats. Therefore, it can be concluded that 3-week physical exercise significantly increased the striatal dopamine and improved anxiety-like behavior by inhibiting the increase of dopamine during acute cerebral ischemia and suppressing the decrease of dopamine after 24 hours and 7 days cerebral ischemia.

## 1. Introduction

Cerebral ischemia is considered among one of the most fatal diseases, which exhibits attributes of higher morbidity, mortality, and disability [1, 2]. Global cerebral ischemia, which normally occurs after a cardiac arrest, can cause selective neuronal cell death, and, as a result, it causes disability and dementia [3, 4]. 2-VO vascular block (2-VO), an animal model made by permanently ligating the bilateral common carotid arteries in rats, can cause a chronic state of cerebral hypoperfusion after ligation, thus producing ischemic and hypoxic damage to brain tissue, which can better simulate human cerebrovascular disease and dementia in the acute ischemic and chronic hypoperfusion periods [5, 6]. At the same time, the 2-VO method produced a whole brain ischemia model in rats without apparent motor system damage, with high survival rate, good repeatability and stability, simple operation, less trauma to the animals, easy recovery, and easy to eliminate the influence of other factors on anxiety-like behavioral experiments in rats [7, 8]. Meanwhile, it has been demonstrated that the functional symptoms of neural damage occur from several hours to days after cerebral ischemia [9]. Furthermore, it can also lead to cognitive and emotional deficits in patients or in experimental animals [10, 11].

Previous reports suggested that cerebral ischemia rats showed increased anxiety-like behavior, activity level, and habituation shortfall [12]. Dopamine levels in specific brain regions have been found to be positively correlated with increased anxiety-like behavior [13]. Several reports support this concept. For example, in vitro study it has been appeared that the slice in the nucleus accumbens of social isolation rearing rats after electrical stimulation resulted in lasting increased anxiety-like behavior, dopamine released, and dopamine transporter activity [9]. While selective dopamine depletion within the prefrontal cortex has been done by 6-hydroxydopamine injection, it could remarkably increase anxiety-like behavior in rats [14]. Dopamine is a well-known catecholaminergic neurotransmitter in the brain that plays an important role in memory processes and emotional aspects [15], particularly through the interconnection of the striatum and the prefrontal cortex [16]. The striatum is one of the most important nuclei of the basal ganglia; its function is vital for motor control, cognition, stimulus-response learning, action selection, and emotional reconciliation [17]. Furthermore, the striatum is known to be one of the core regions susceptible to ischemic insult [18]. Numerous studies have shown that cerebral ischemia can cause dopamine release in the striatum [19, 20]. In addition, in vivo imaging studies have been observed that striatal dopamine is a key player in anxiety disorders [21, 22]. Pharmacological research has shown that medicine therapy could alleviate symptoms of anxiety by decreasing excessive dopamine accumulation during the acute cerebral ischemia/reperfusion [14]. However, early efforts have been made to understand regulation of anxiety that are closely related to striatal dopamine synthesis capacity [9, 12]. Further studies have shown that moderate treadmill exercise before cerebral ischemia can prevent oxidative stress-prompted anxiety-like behavior in rats [23]. When compared to treadmill exercise, the advantage of voluntary exercise is that it is an active and spontaneous exercise that can significantly stimulate the autonomic activity of the rat and significantly increased the level of neurotrophic factor in rat brain and enhanced neuroplasticity [24]. Although it has been well documented that striatal dopamine released induced by ischemic brain injury has a closely related anxiety-like behavior, whether pre-ischemia wheel running has anxiolytic effects by regulating level of the dopamine in the striatum is currently unknown.

As a result, the purpose of this study was to investigate the effect of voluntary wheel running on striatal dopamine levels and anxiety-like behavior in rats following cerebral ischemia.

## 2. Materials and Methods

### 2.1. Animals and Training

Male Sprague-Dawley rats (3 months old, weighting 300 ± 10 g) were procured from Peking University's Experimental Animal Center and randomly divided into following 4 groups: Control group (C group), sham group (S group), ischemia group (I group), and 3 weeks voluntary wheel running before ischemia group (3RI group). The rats in the 3RI groups were placed in a voluntary running wheel (wheel circumference, 100 cm, Harvard Apparatus) for three weeks to exercise. The groups C, S, and I were also assigned to the reorganized voluntary running wheel (running wheel were fixed). The running wheel was equipped with a magnetic counter to track wheel revolutions [25]. The distance was calculated by multiplying the number of wheel revolutions by the circumference of the wheel. In accordance with previous research, the rats primarily exercised at night from 19 : 00 to 7 : 00, covering approximately 5000 meters per week [26]. According to our experiments, the distance of animals exercised autonomously were about 1250 m in the first three days, and the average exercise distance were about 400 m per day. These rats that exercise distance less than 200 m per day were excluded. Throughout the experiment, there were 3 animals that were not able to keep up with the mentioned conditions. It was decided not to collect the data of these three rats, and it was chosen to select new rats to replace them. Then, the rats in 3RI and I group were received bilateral carotid artery ligation (2-VO) operation. In the S group, only the bilateral common carotid arteries were exposed without ligation. All rats were housed in 12-hour light/dark cycles with plenty of food and water. All procedures and protocols were approved by the institutional animal ethical committee of the Capital University of Physical Education and Sports (2020A53). The experiment was carried out in accordance with approved guidelines of Good Treatment of Laboratory Animals issued by the Ministry of Science and Technology of China.

### 2.2. Surgical Procedure

The stereotaxic surgery method was based on previous research [27]. The rats were placed in a stereotaxic frame with the incisor-bar set at 3.3 mm below the interaural line for the flat skull position after being anesthetized with 8% emulsified isoflurane (0.55 ml/kg, intraperitoneal injection). According to standard stereotaxic procedures, a guide cannula was lowered into the right striatum (anteroposterior = 0 mm, mediolateral = 3.0 mm, dorsoventral = 4 mm, relative to bregma) [28]. Three screws were inserted into the skull around the cannula and secured with dental acrylic.

The bilateral common carotid arteries (BCCAs) were ligated in the two-vessel occlusion (2-VO) ischemia models using the methods previously described [29, 30]. In brief, the rats were anesthetized intraperitoneally with 10% chloral hydrate (350 mg/kg i.p.), fixed supine, disinfected with anterior cervical debridement, incised along the middle of the neck, and the double common carotid arteries were isolated and double ligated with No.1 surgical wire. At the same time, the cervical sympathetic nerve and vagus nerve were avoided, and the rats' anal temperature was kept at 37 ± 0.5°C during the operation, as was spontaneous respiration.

### 2.3. In Vivo Microdialysis and High Performance Liquid Chromatography

In vivo microdialysis was carried out by inserting a microdialysis probe (CMA/12, CMA/microdialysis AB, membrane length = 4 mm, Stockholm, Sweden) through the guide cannula and continuously perfusing with artificial cerebrospinal fluid (126 mM NaCl, 2.4 mM KCl, 1.1 mM CaCl_2_, 0.85 mM MgCl_2_, 27.5 mM NaHCO_3_, 0.5 mM Na_2_SO_4_, 0.5 mM KH_2_PO_4_, pH = 7.0) at a flow rate of 2 L/min driven by a microinjection pump (CMA/100, CMA Microdialysis AB, Stockholm, Sweden).

After 90 minutes, samples were collected in a 0.2 ml vial, and microdialysis was performed in the striatum at a flow rate of 2 *μ*L/min for a continuous 60 minutes. Samples were taken at two different times, one before and one after ligation. In addition, we used an animal awake activity device to collect 120 *μ*L of striatal dopamine of rats 24 hours after cerebral ischemia. The samples were analyzed for DA content in an HPLC system equipped with an isocratic pump (Waters Corporation, Milford, MA, USA, Waters 515, flow 1 ml/min), an RP-18 column (Waters Corporation, Milford, MA, USA, Xterra, 5 *μ*M particle size, 2.1 × 100 mm, Waters), and an amperometric detector (BAS Inc., West Lafayette, IN, USA, LC-4B, oxidation potential 0.5 V). The DA elutions were completed in all of these conditions in 4.5 minutes.

### 2.4. Behavioral Testing

The elevated plus maze (EPM) was used to analyze anxiety-like behavior by determining animals' emotional response to external stressful stimuli [31]. The EPM was made of black polypropylene and had two opposing open arms (25 × 8 cm), two opposing closed arms (25 × 8 × 20 cm), and a central platform (8 × 8 × 8 cm) shaped like a cross. The maze was 50 cm above the ground. Individual rats were placed in the center, their heads pointing toward one of the closed arms and given 5 minutes to explore the arena. All four paws entering an arm were defined as an open or closed arms entry. The open-field test (OF) was used to determine animals' spontaneous locomotor activity [32]. The OF test was conducted after EPM test. The interval between the two experiments was 1 hour. In standard room lighting conditions, the OF was performed in a 50 × 50 cm open field surrounded by 50 cm high walled Plexiglas chambers. Individual rats were placed in the center and given 5 minutes to explore the arena. Both experiments were tested at 24 hours and 7 days after cerebral ischemia.

To avoid external distractions, the behavioral tests were conducted in an isolated behavioral testing room within the animal facility. Animal behavior was observed by investigators via a video monitor in another room. Shanghai Mobile Datum Information Technology Co., Ltd. provided the apparatus and analysis software used in the behavioral tests. Rats were housed in the testing room for at least 1 hour before the experiments to facilitate adaptation to the experimental environment. The animals were uninformed about the test situation and were only used once [33]. Alike the other behavior test, the EPM and OF were cleaned with a solution of 75% ethyl alcohol after testing.

### 2.5. Gait Analysis in Rats

In order to eliminate the effect of changes in the locomotor ability of rats before and after cerebral ischemia on subsequent behavioral experiments, the present experiment was conducted to evaluate the locomotor ability of experimental rats using a previously reported method. The gait parameters were measured before and after the experiment by applying different color dyes to the front and hind paws of the rats when they were freely moving [34]. The gait angle of right foreleg (GARF), the vertical distance between the anterior and posterior step lines on both sides of the foreleg (track width of foreleg (TWF), the horizontal distance between the midpoint of the horizontal line of two consecutive steps of the right foreleg and the midpoint of the horizontal line of two consecutive steps of the hind limb (foot base of right foreleg, FBRF), and the horizontal distance of the line of the right foreleg (right foot base, RFB) were measured three times consecutively. The above parameters were statistically analyzed.

### 2.6. Statistical Analysis

Data were analyzed by SPSS25.0 (SPSS Inc., Chicago, IL, USA), and the results were expressed as means ± standard deviation (SD), and all data showed normal distribution. Repeated measures ANOVA was used to compare dopamine in different groups at various time points. Differences in the behavior and gait data were analyzed using one-way ANOVA, followed by the Bonferroni test for intergroup comparisons. A *P* value < 0.05 was considered statistically significant.

## 3. Results

### 3.1. Elevated plus Maze

**Figure 1:**
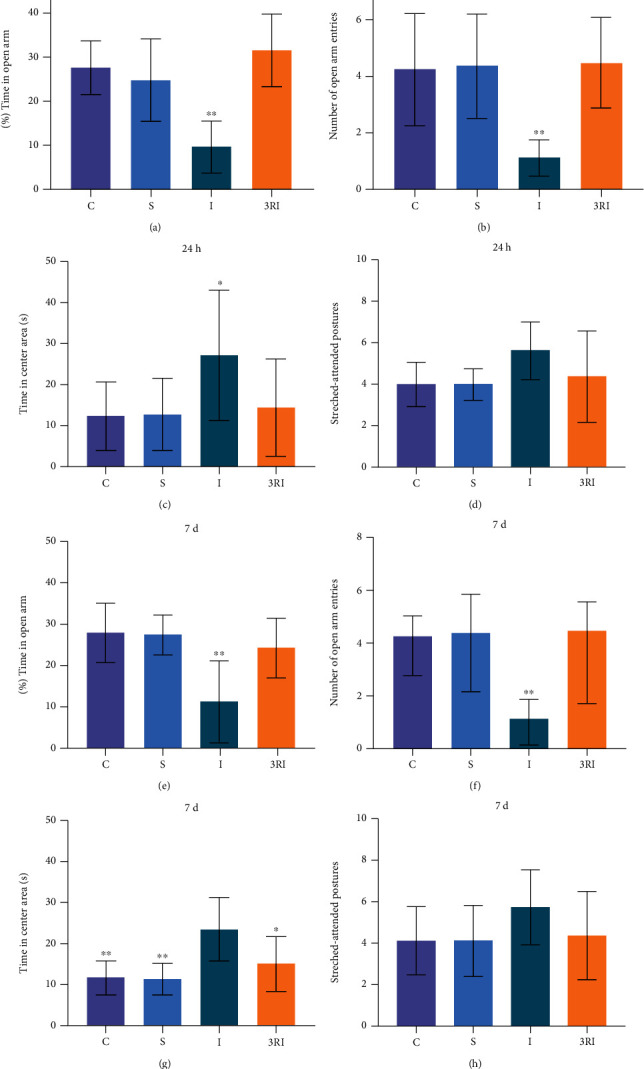
(a) The percent time of open arms in each group at 24 hours after ischemia. ^∗∗^*P* < 0.01 compared with I group; (b) the entrance numbers of open arms in each group. ^∗∗^*P* < 0.01 compared with I group; (c) the time in center area in each group. ^∗^*P* < 0.05 compared with I group; (d) the stretched-attended postures of 4 groups after ischemia. (e) the percent time of open arms in each group at 7 days after ischemia. ^∗∗^*P* < 0.01 compared with I group; (f) the entrance numbers of open arms in each group. ^∗∗^*P* < 0.01 compared with I group; (g) the time in center area in each group. ^∗^*P* < 0.05 compared with I group; (h) the stretched-attended postures of 4 groups after ischemia.

Figure 1(a) shows that the percent time of open arms in each group after ischemia. The percent time in the 4 groups had significant difference (F (3, 28) = 12.941, *P* = 0.000017). Bonferroni multiple comparison post hoc test showed that the percent time of C, S and 3RI group was significantly longer when compared with I group (*P* < 0.01). There was no significant difference among C, S and 3RI group (*P* > 0.05).  Figure 1(b) showed the entrance numbers of open arms in each group after ischemia. The entrance numbers in the 4 groups had significant difference (F (3, 28) = 8.219, *P* = 0.000444). Bonferroni multiple comparison post hoc test showed that the entrance numbers of C, S, and 3RI group were significantly greater when compared with I group (*P* < 0.01). There was no significant difference among C, S, and 3RI group (*P* > 0.05).  Figure 1(c) showed that the time in center area of each group after ischemia. The time in center area in the 4 groups had significant difference (F (3, 28) = 3.359, P = 0.033). Bonferroni multiple comparison post hoc test showed that the entrance numbers of C, S, and 3RI group were significantly shorter when compared with I group (*P* < 0.05). There was no significant difference among C, S, and 3RI group (*P* > 0.05).  Figure 1(d) shows that stretched-attended postures of each group after ischemia. The stretched-attended postures had no significant difference in the 4 groups (*F* (3, 28) = 2.226, *P* = 0.107). Although the stretched-attended postures of I group were increased, there were no significant difference when compared with C, S, and 3RI group (*P* > 0.05).

Figure 1(e) shows that the percent time of open arms in each group after ischemia. The percent time in the 4 groups had significant difference (*F* (3, 28) = 9.157, *P* = 0.00022). Bonferroni multiple comparison post hoc test showed that the percent time of C, S, and 3RI group were significantly longer when compare with I group (*P* < 0.01). There was no significant difference among C, S, and 3RI group (*P* > 0.05).  Figure 1(f) showed the entrance numbers of open arms in each group after ischemia. The entrance numbers in the 4 groups had significant difference (*F* (3, 28) = 7.551, *P* = 0.00075). Bonferroni multiple comparison post hoc test showed that the entrance numbers of C, S, and 3RI group were significantly greater when compare with I group (*P* < 0.01). There was no significant difference among C, S, and 3RI group (*P* > 0.05).  Figure 1(g) showed the time in center area of each group after ischemia. The time in center area in the 4 groups had significant difference (F(3, 28) = 7.192, P = 0.0011). Bonferroni multiple comparison post hoc test showed that the entrance numbers of C, S and 3RI group were significantly shorter when compare with I group (*P* < 0.05). There was no significant difference among C, S, and 3RI group (*P* > 0.05).  Figure 1(h) shows that stretched-attended postures of each group after ischemia. The stretched-attended postures were no significant difference in the 4 groups (*F* (3, 28) = 1.432, *P* = 0.254). Although the stretched-attended postures of I group were increased, there were no significant difference when compared with C, S, and 3RI group (*P* > 0.05).

### 3.2. Open Field Test

**Figure 2:**
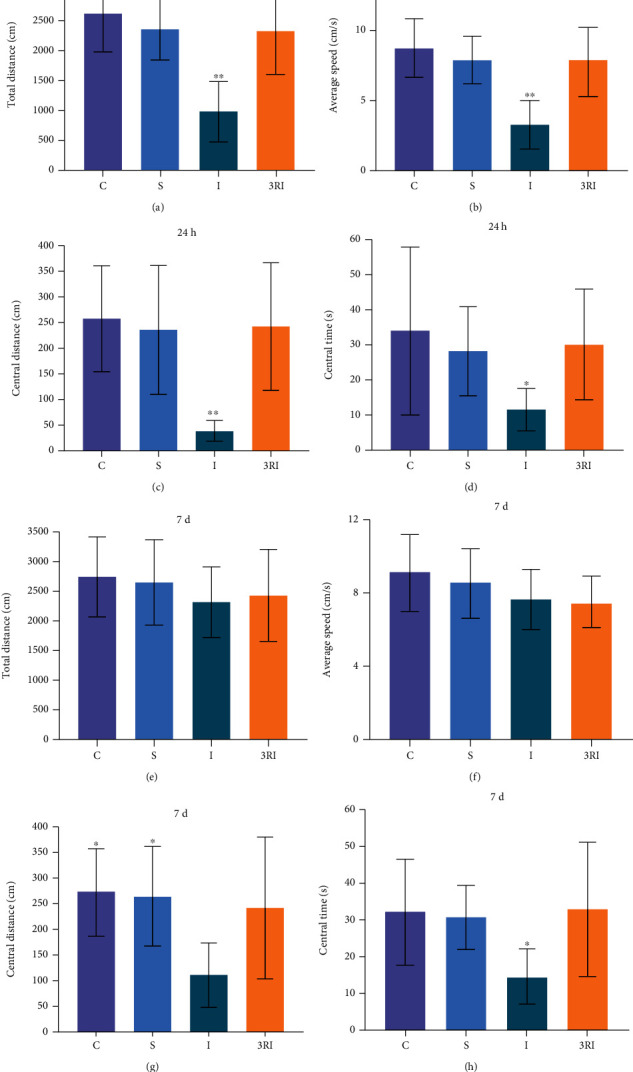
(a) The total distance of 4 groups in open field test at 24 hours after ischemia. ^∗∗^*P* < 0.01 compared with I group. (b) The average speed of 4 groups in open field test. ^∗∗^*P* < 0.01 compared with I group. (c) The central distance of 4 groups in open field test. ^∗^*P* < 0.05 compared with I group. (d) The central time of 4 groups in open field test. ^∗∗^*P* < 0.05 compared with I group. (e) The total distance of 4 groups in open field test. (f) The average speed of 4 groups in open field test. (g) The central distance of 4 groups in open field test. ^∗^*P* < 0.05 compared with I group. (h) The central time of 4 groups in open field test. ^∗^*P* < 0.05 compared with I group.

Figure 2(a) shows that the total distance of each rat groups in open field test. The total distance in the 4 groups had significant difference (F (3, 28) = 11.851, *P* = 0.000035). Bonferroni multiple comparison post hoc test showed that the total distance of C, S, and 3RI group were significantly longer when compared with I group (*P* < 0.01). There were no significant differences among C, S, and 3RI group (*P* > 0.05).

Figure 2(b) shows the average speed of each group in open field test. The average speed in the 4 groups had a significant difference (F (3, 28) = 11.851, *P* = 0.000035). Bonferroni multiple comparison post hoc test showed that the average speeds of C, S, and 3RI group were significantly faster when compared with I group (*P* < 0.01). There were no significant differences among C, S, and 3RI group (*P* > 0.05).

Figure 2(c) shows that the central distance of 4 groups in open field test. The central distance in the 4 groups had significant difference (*F* (3, 28) = 6.774, *P* = 0.001). Bonferroni multiple comparison post hoc test showed that the central distances of C, S, and 3RI group were significantly longer when compared with I group (*P* < 0.01). There were no significant differences among C, S, and 3RI group (*P* > 0.05).

Figure 2(d) shows the central time of 4 groups in open field test. The central time in the 4 groups had significant difference (*F* (3, 28) = 3.132, *P* = 0.041).Bonferroni multiple comparison post hoc test showed that the central time of C, S, and 3RI group was significantly longer when compared with I group (*P* < 0.05). There were no significant differences among C, S, and 3RI group (*P* > 0.05).

Figure 2(e) shows the total distance of each rat groups in open field test. The total distance in the 4 groups had significant difference (*F* (3, 28) = 0.629, *P* = 0.603). Bonferroni multiple comparison post hoc test showed that the total distance of C, S, I, and 3RI group had no significant difference (*P* > 0.05).

Figure 2(f) shows the average speed of each group in open field test. The average speed in the 4 groups had significant difference (F (3, 28) = 1.513, *P* = 0.233). Bonferroni multiple comparison post hoc test showed that the average speed of C, S, I, and 3RI group were no significant difference (*P* > 0.05).

Figure 2(g) shows the central distance of 4 groups in open field test. The central distance in the 4 groups had significant difference (*F* (3, 28) = 4.511, *P* = 0.011). Bonferroni multiple comparison post hoc test showed that the central distances of C and S group were significantly longer when compared with I group (*P* < 0.01). There were no significant differences among C, S, and 3RI group (*P* > 0.05).

Figure 2(h) shows the central time of 4 groups in open field test. The central time in the 4 groups had significant difference (*F* (3, 28) = 3.559, *P* = 0.027). Bonferroni multiple comparison post hoc test showed that the central time of C, S, and 3RI group were significantly longer when compare with I group (*P* < 0.05). There were no significant differences among C, S, and 3RI group (*P* > 0.05).

### 3.3. Gait Analysis in Rats

**Figure 3:**
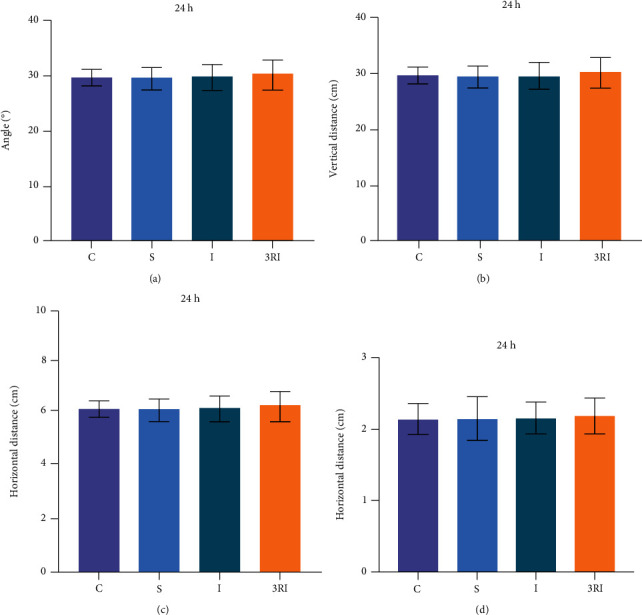
The gait analysis of 4 groups.

Figures 3(a)–3(d) show gait parameters of each rat groups. The GARF (*F* (3, 28) = 0.165, *P* = 0.919), TWF (*F* (3, 28) = 0.197, *P* = 0.897), FBRF (*F* (3, 28) = 0.228, *P* = 0.876), and RFB (*F* (3, 28) = 0.039, *P* = 0.99) had no significant difference among 4 groups.

### 3.4. The Striatal Dopamine Level in each Group

**Figure 4:**
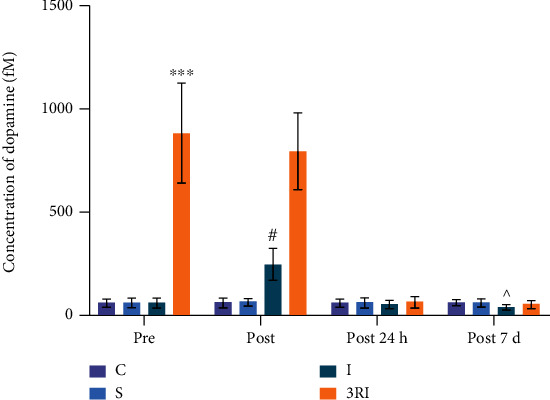
The changes of striatal dopamine in each group at 4 points in time. ^∗∗∗^*P* < 0.001 compared with I and S group before ischemia. ^#^*P* < 0.05 compared with I group before ischemia. ^∧^*P* < 0.05 compared with S group at 7 days after ischemia.

Figure 4 shows the changes in striatal dopamine in each group before ischemia, during the acute phase of ischemia, and 24 hours after ischemia. A 4×4 repeated measures ANOVA was performed on dopamine, with ischemia as a between-group factor and time (before, acute phase, 24 hours, 7 days) as a within-group factor. These results showed a significant main effect of ischemia (*F* (3, 28) = 104.599, *P* = 0.000023, *η*^2^ = 0.918) and a significant main effect of time (Greenhouse-Geisser adjusted F (1.771, 49.6) = 103.714, *P* = 0.00004, *η*^2^ = 0.787), for dopamine, which was significantly higher in the ischemia than non-ischemia group. There was also a significant ischemia × time interaction effect (Greenhouse-Geisser adjusted F (5.314, 49.6) = 77.153, *P* = 0.00012, *η*^2^ = 0.892). When compared with C and S group, the extracellular dopamine level had no significantly difference at the four time points (*P* > 0.05). When compared with dopamine of I group, the dopamine of 3RI group has significant difference before ischemia (*P* < 0.001). The extracellular dopamine of I group has significantly increased (*P* < 0.05), and the extracellular dopamine of 3RI group has no significant difference during the acute phase of ischemia when comparing pre-ischemia (*P* > 0.05). At 24 hours after ischemia, striatal dopamine returned to pre-ischemic levels in 3RI group (*P* > 0.05). Striatal dopamine in I group was smaller than pre-ischemic levels at 7 days (*P* < 0.05).

### 3.5. Voluntary Exercise Behavior

Average running distance of rats in this experiment was 679 m each night. The distance increased rapidly over the first week and reached the maximum mean distance of 885 m on 7th night and then remained around 726 m. The mean distance of the rats in the 3 weeks was about 14265 m.

## 4. Discussion

Cerebral ischemia in humans is a common and frequently-occurring disease, which leads to limb movement disorders, cognitive dysfunction, and emotional disorders of ischemia patients [35]. New studies revealed that cerebral ischemia can give rise to emotional impairment resulted by increased anxiety-like behavior [36, 37]. Studies in the past have revealed that high-anxiety animals were less exploration behavior in the open arms of elevated plus maze and central zone of open field test [38]. Thus, elevated plus maze and open field test are used in evaluation of anxiety-like behavior [39, 40]. As shown in Figures 1 and 2, the percent time and entrance number of open arms in I group were significant less than C, S, and 3RI group. Besides, the time in center area in I group were significant more than C, S, and 3RI group. And the central time and central distance of open field in I group were significant less than C, S, and 3RI group, either. This result indicated that 24 hours cerebral ischemia can lead to anxiety-like behavior in rats. These findings are in accordance with the previous study. The findings of the previous studies have revealed that the rats could develop anxiety-like behavior after global cerebral ischemia [41]. Therefore, this experiment successfully established model of anxiety in rat after global cerebral ischemia. In addition, this study also found that the average speed and total distance of open field in I group were significant less than S and 3RI group. It was believed that the animals may develop depression-like behavior, which is not in accordance with previous studies and may be due to the different modelling methods used, as the previous study only ligated the common carotid arteries bilaterally for 10 minutes [42], whereas this experiment used a permanent arterial ligation. Therefore, whether depression-like behavior occurs in rats after permanent ligation of the bilateral common carotid artery and whether exercise obstructs this behavior requires further study. Besides, our study found that rats also showed significant anxiety-like behavior on day 7 after cerebral ischemia, and this study is consistent with previous studies. Previous study found that rats exhibited significant anxiety-like behavior after 1 week of cerebral ischemia [43]. Furthermore, in order to eliminate the influence of non-specific motor problems on the findings of this experiment, gait and motor coordination evaluation was carried out on each group of animals, and the findings revealed that there were no significant differences among the four groups (Figure 3).

The findings of this experiment also revealed that the content of striatal dopamine was notably increased after ischemia; there was significant difference between before and acute ischemia in I group (Figure 4). The results were in accordance with previous research. It has been found that the striatal dopamine content increased significantly, rising to 150% of the baseline level at 20 minutes, and the dopamine level in the striatum at 120 minutes after ischemia was significantly higher than that before ischemia [44]. The findings of the previous studies have shown that cerebral ischemia instigates a neurotoxic cascade resulting in various biochemical and metabolic disturbances such as excessive dopamine accumulation in striatum. A substantial body of evidence has progressively researched the mechanism of deleterious effect of excessive dopamine on neurons. First, oxidation of large amounts of dopamine promotes the generation of free radicals, in particular, in regions of the brain such as the striatum [45]. Second, the oxidized dopamine can form covalent bonds, which may lead to modification of protein structure and function and cause further tissue damage [46]. Third, excessive dopamine can also react with hydroxyl radicals to generate the more dopaminergic neurotoxin 6-hydroxydopamine [47]. These results suggest that dopamine accumulation may be closely related to acute cerebral ischemic damage. Therefore, attenuation or prevention of striatal dopamine accumulation during the acute cerebral ischemia may reduce ischemia-induced impairments in anxiety-like behavior.

In addition, our study found that rats had significantly lower striatal dopamine levels than controls on day 7 after cerebral ischemia. Consistent with our findings, recent studies have suggested that the depletion of dopamine in the striatum can induce anxiety-like behavior in low exploratory rats [48]. Our preliminary experimental study also found that anxiety-like behavior instigated by molar loss was strongly associated with a decrease in striatal dopamine levels [49]. Furthermore, quetiapine effectively attenuated anxiety-like behavior and neurotoxicity in dopaminergic terminals [50]. Thus, it is reasonable to suggest that the decrease of striatal dopamine level in chronic cerebral ischemia can be one of the important reasons of anxiety-like behavior in cerebral ischemia rats.

**Figure 5:**
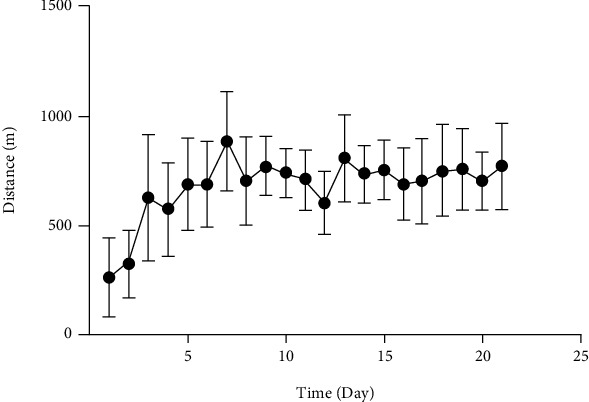
Average wheel running distance over 21 days.

In recent years, many researches have committed on the treatment methods and mechanisms of cerebral ischemia [51, 52], However, therapeutic strategies for the anxiety caused by cerebral ischemia have not been well researched. Studies showed that aerobic exercise attenuates ischemia-induced memory impairment by enhancing cell proliferation and suppressing neuronal apoptosis [53]. Besides, some research showed that exercise training had antidepressant and anxiolytic effects and protected against harmful consequences of stress [54, 55]. The exercise methods of involuntary, forced, and voluntary exercise were extensively used in animals in this research [56]. Further studies indicated that voluntary wheel running can be one of the best ways to promote cognition and emotion [57, 58]. Meanwhile, some researches showed that 3 weeks prerequisite exercise improves behavioral functions following transient cerebral ischemia in rats [59]. Thus, the present study selected 3 weeks voluntary wheel running as preventative approach. As shown in Figure 5, we found that the rats in this experiment exhibited the similar exercise tendency by recording the daily running distance of the rats; therefore, the exercise protocol used in this experiment was proved effective [60]. Researchers have shown that exercise can enhance blood flow [61], oxygenation [62], and levels of neurotrophic factor [63] and vascular endothelial growth factor [64] in the brain that provided neuroprotection to the brain under ischemic conditions. Further, our research demonstrate that 3 weeks voluntary wheel running can inhibit dopamine levels during acute cerebral ischemia. Thus, it is viable to suggest that maintaining stable dopamine levels during acute cerebral ischemia can be the reasons to induce that voluntary wheel running improve anxiety-like behavior after cerebral ischemia in rats.

It is worth to mention that 3 weeks voluntary wheel running not only remarkably improved the anxiety of cerebral ischemia rats, but also notably increased the dopamine level in striatum. The experimental study found that the striatal dopamine in 3RI group was crucially higher than I and S group before and after ischemia; its maximum value reached about 15 and 12 times of I and S group before and after ischemia. Reference to the literature on exercise-induced changes in striatal dopamine levels is scarce. Some past studies observed that striatal dopamine content of endurance training and sham group were significantly increased and remained at a basic level about 150% in the 60-minute training course and about 3 hours after the training [65, 66]. This study revealed that striatal dopamine levels of rats increased significantly after voluntary running wheel exercise and returned to basal levels 24 h after cessation of exercise. These findings suggested that exercise stimulated the release of dopamine in striatum and the increase of striatal dopamine will return to the base level over a period of time, which was consistent with the results of this study.

Although we found that 3 weeks voluntary wheel running has the tendency to improve the anxiety-like behavior induced by cerebral ischemia and increased the striatal dopamine, it is unsured whether this releaving of anxiety after exercise is directly induced by the dopamine releasing or by other exercise-related neurochemical modification. It is required to further study addressing at correlation between dopaminergic neural circuit effected by exercise and anxiety-related changes of dopamine level by neuropsychopharmacology experiments.

## 5. Conclusions

3 weeks exercise can outstandingly increase the striatal dopamine level before ischemia and improve the anxiety-like behavior in rats of cerebral ischemia by inhibiting the increase the striatal dopamine level during acute cerebral ischemia and suppressing the decrease of dopamine after 24 hours cerebral ischemia.

## Data Availability

Data are available by contacting the corresponding author.
